# Gene and cytokine profile analysis of macrolide-resistant *Mycoplasma pneumoniae* infection in Fukuoka, Japan

**DOI:** 10.1186/1471-2334-13-591

**Published:** 2013-12-16

**Authors:** Kentaro Matsuda, Mitsuo Narita, Nobuyuki Sera, Eriko Maeda, Hideaki Yoshitomi, Hitomi Ohya, Yuko Araki, Tatsuyuki Kakuma, Atsushi Fukuoh, Kenji Matsumoto

**Affiliations:** 1 Matsuda Children’s Clinic, Fukuoka, Japan; 2 Department of Allergy and Immunology, National Research Institute for Child Health and Development, Setagaya, Tokyo, Japan; 3 Department of Pediatrics, Sapporo Tokushukai Hospital, Sapporo, Hokkaido, Japan; 4 Division of Virology, Fukuoka Institute of Health and Environmental Sciences, Dazaifu, Fukuoka, Japan; 5 Division of Microbilogy, Kanagawa Prefectural Institute of Public Health, Chigasaki, Kanagawa, Japan; 6 Biostatics Center, Kurume University School of Medicine, Kurume, Fukuoka, Japan; 7 Department of Medical Laboratory Science, Junshin Gakuen University, Fukuoka, Fukuoka, Japan

**Keywords:** *Mycoplasma pneumoniae*, Macrolide resistant, A2063T, Inflammatory cytokine, Predictive factors

## Abstract

**Background:**

Recent epidemiologic data suggest that the prevalence of macrolide resistant *Mycoplasma pneumoniae* (MR-*M. pneumoniae*) is increasing rapidly worldwide. This study assessed the present status of *M. pneumoniae* infection in Japan and clinical end-points to distinguish children with MR-*M. pneumoniae*.

**Methods:**

During an outbreak of *M. pneumoniae* infections in Fukuoka, Japan in 2010–11, a total of 105 children with clinically suspected *M. pneumoniae* infection were enrolled. *M. pneumoniae* was analyzed for macrolide resistance in domain V of the 23S rRNA gene. Sixty -five patients with PCR positive for *M. pneumoniae* were analyzed with regard to clinical symptoms, efficacy of several antimicrobial agents and several laboratory data.

**Results:**

Causative pathogens were detected in 81.0% (85 of 105) and *M. pneumoniae* was identified 61.9% (65 of 105). The resistance rate of *M. pneumoniae* was 89.2% (58 of 65) in this general pediatric outpatient setting. Patients infected with MR-*M. pneumoniae* showed longer times to resolution of fever and required frequent changes of the initially prescribed macrolide to another antimicrobial agent. We observed three different genotypes of *M. pneumoniae* including the rarely reported A2063T mutation (A2063G: 31 strains, A2063T: 27 strains, no mutation: 7 strains). Drug susceptibility testing showed different antimicrobial susceptibility profiles for each genotype. Serum IFN-gamma, IL-6 and IP-10 levels were higher in patients with MR-genotypes than in those infected with no-mutation strains (p < 0.001).

**Conclusions:**

Macrolide resistance is more common than previously thought and a small epidemic of rarely reported A2063T mutation was observed in Fukuoka, Japan. Furthermore our results reveal the possibility that levels of certain inflammatory cytokines may be a candidate to predict MR-*M.pneumoniae* infection.

## Background

*Mycoplasma pneumoniae* (*M. pneumoniae*) is a common bacterial cause of upper and lower respiratory tract infections in children and adolescents. Recent advances in antimicrobial agents, including macrolide antibiotics [1], tetracyclines and fluoroquinolones, have enabled its treatment on an outpatient basis for most children with community-acquired pneumonia. Since the time when macrolide resistant (MR) *M. pneumoniae* was first isolated from a pediatric patient in 2000 [2], the drug resistance trend has been documented all over the world [3-10], especially in children. Extremely high rates of macrolide resistance have been reported mainly in Asian countries [3,4]. According to the continuous surveillance in Japan, the frequency of MR-*M. pneumoniae* cases has increased year by year, and nowadays >80% of *M. pneumoniae* isolates were shown to be macrolide resistant [4]. However, most of these reports included the data in secondary or tertiary care facilities; thus very little information is currently available on the prevalence of MR-*M. pneumoniae* infection, especially in outpatient settings. Macrolide-resistant genotypes can be defined by the detection of specific point mutations in the domain V of the single-operon derived 23S rRNA gene of M. pneumoniae. While most frequent mutations that induce high levels of macrolide-resistance included an A-to-G transition at position 2063 or 2064 [11,12], recent epidemiologic studies have suggested that distribution of MR-*M. pneumoniae* genotypes has been changing [4,13]. The primary objective of our study was to clarify the present status of MR-*M. pneumoniae*. For the present, it is difficult to make an early identification of which children being infected by MR-*M. pneumoniae*. Thus, secondly, we evaluated children with *M. pneumoniae* infections with regard to clinical symptoms, efficacy of several antimicrobial agents and several laboratory data to investigate the predictive factors of MR-*M. pneumoniae* infections.

## Methods

### Subjects

Between September 2010 and December 2011, a total of 105 patients with clinically suspected *M. pneumoniae* infection were recruited from Matsuda Children’s Clinic (Onojo-city, Fukuoka Prefecture) after informed consent was obtained from both the children and their parents. Nasopharyngeal swab specimens and blood samples were obtained from all patients at the time of enrollment, and the throat swabs were sent to the Laboratory of Virology, Fukuoka Institute of Health and Environmental Sciences. Laboratory-confirmed *M. pneumoniae* infection was defined as detection of *M. pneumoniae* DNA from the throat swab by PCR methods. Multiplex PCR was carried out as described previously [14,15]. *M. pneumoniae*, rhinoviruses (RV), respiratory syncytial virus (RSV), coronaviruses, human metapneumovirus (HMPV), human bocavirus, influenza A, and B viruses, adenovirus and parainfluenza virus (PIV 1–4). Ultimately, data on 65 children (33 girls, 32 boys; mean age 6.1 years) with positive for *M. pneumoniae* were analyzed. This study was approved by the Ethical Committee of Kurume University School of Medicine and the National Research Institute for Child Health and Development.

### Measurement of biologic markers

To evaluate the disease activity of *M. pneumoniae* infection, we performed general blood tests, including white blood cell (WBC) counts, C-reactive protein (CRP), erythrocyte sedimentation rate (ESR) at the time of throat swab collection. Serum concentrations of chemokines and cytokines were measured using Luminex xMAP technology (Milliplex MAP kits; Millipore, Billerica, MA, USA) according to the manufacturer’s instructions. The detection limits are 2.0, 12.5, 1.13, 8.0, and 1.21 pg/mL for IL-8, IL-18, IFN- gamma, IP-10 and IL-6, respectively.

### Measurement of clinical efficacy

Clinical records of the patients with *M. pneumoniae* infection were examined retrospectively. Precise quantitative data such as total duration of fever and the usage of antibiotics which were obtained from the clinical charts and the clinical courses of patients infected by each of the three different genotypes (A2063G, A2063T, and no mutation) were compared. A febrile day was defined as a day during which the body temperature exceeded 37.5°C. As an indicator of the severity of mycoplasma infection, the number of patients requiring hospitalization was also assessed. The chemotherapeutical agent was often changed from a macrolide to minocycline (MIN) or tosufloxacin (TFX) according to clinical conditions such as persistent fever and cough as well as chest X-ray findings. The attending physician (K. Matsuda) had no information about the susceptibility to *M. pneumoniae* at the time of clinical decision-making.

### PCR amplification and sequencing

The mutations associated with resistance to macrolides were detected by sequencing the targeted domain V region of the 23S rRNA gene as described by Matsuoka et al. (11) using the following primers for domain V of 23S rRNA (Forward:5′-GCAGTGAAGAAGAACGAGGGG-3′, Reverse:5′-GTCCTCGCTTCGGTCCTCTCG-3′) which were synthesized as reported by Lucier et al. [16]. The sequences were aligned using the ClustalW option within the software Molecular Evolutionary Genetics Analysis (MEGA) 4.0.2 (http://www.megasoftware.net/) and compared to the registered sequence of *M. pneumoniae* M129 (accession No. ×68422).

### Minimal inhibitory concentration determination

Minimal inhibitory concentrations (MICs) of antibiotics were determined by a broth microdilution method based on the National Committee for Clinical Laboratory Standards (Clinical and Laboratory Standards Institute) as described elsewhere [2,11].

The following antibiotics were tested: erythromycin (ERY), clarithromycin (CLR), azithromycin (AZM), josamycin (JOS), rokitamycin (RKI), clindamycin (CLI), tetracycline (TET), MIN, levofloxacin (LVX), ciprofloxacin (CPX), TFX, sparfloxacin (SPX) and gatifloxacin (GFX). Briefly, serial twofold dilutions of antibiotics were prepared in the Mycoplasma broth base inoculated by 10^4^ to 10^5^ CFU/ml of *M. pneumoniae* in 96-well microplates and incubated at 37°C. The MIC was determined as the lowest concentration of antimicrobial agent at which the color of the control medium changed. The degree of resistance was evaluated according to the Japanese standard (</= 4 μg/mL; susceptible, 8 μg/mL; intermediate, > /= 16 μg/mL; resistant, for ERY, CLR, AZM, JOS, RKI, CLI) [17].

### Statistical analysis

Statistical analysis was performed using the computing environment R (R Development Core Team, 2012). The data in Table 1 are presented as medians (1^st^ and 3^rd^ quartiles). The Kruskal-Wallis test or Fisher’s exact test was used for multiclass comparison (A2063G, A2063T, no mutation). If the results were significant, then the Wilcoxon rank-sum test was used for pairwise comparisons (A2063G vs no mutation, A2063T vs no mutation, and A2063G vs A2063T, respectively). P-values less than 0.05 were regarded as statistically significant.

**Table 1:** **Comparison of clinical characteristics in patients infected by *M. pneumoniae *** **according to the genotypes**

	**A2063G (n = 31)**	**A2063T (n = 27)**	**No mutation (n = 7)**	***p*** *	***P1***	***P2***	***P3***
Age (years)	7.0 (4.0 – 9.5)	7.0 (4.5 – 8.0)	4.0 (3.0 – 7.5)	ns			
Gender (M/F)	14 / 17	15 / 12	3 / 4	ns			
Day exam performed	4 (4 – 6)	5 (4 – 5)	5 ( 4 – 5)	ns			
WBC	5850	6200	6350	ns			
(/μl)	(5050–6250)	(5650–7075)	(5375–6800)				
CRP	1.35	0.90	1.75	ns			
(mg/dL)	(0.90 - 1.95)	(0.53 -2.48)	(0.63 - 4.08)				
ESR	29.0	30.0	34.5	ns			
(mm/hr)	(22.75 - 40.50)	(22.0 - 44.25)	(25.25 - 52.0)				
IL-8	14.2	20.1	29.88	ns			
(pg/mL)	(9.1 - 34.3)	(15.2- 26.0)	(18.4 - 84.8)				
IL-18	457.0	477.7	533.8	ns			
(pg/mL)	(410.2 - 571.6)	(362.5 - 564.2)	(420.9 - 629.8)				
IFN-γ	15.9	18.1	11.5	< 0.001	< 0.001	< 0.001	ns
(pg/mL)	(10.4 – 27.2)	(4.7 – 38.6)	(8.8 – 12.7)				
IP-10	1019	931	643.5	< 0.001	< 0.001	< 0.001	ns
(pg/mL)	(794–1378)	(659 – 1519.5)	(550.7 – 873.0)				
IL-6	6.57	7.79	5.86	< 0.001	< 0.001	< 0.001	ns
(pg/mL)	(2.77 – 13.73)	(3.34 – 16.38)	(3.16 – 11.58)				
Initial antibiotics used							
none	0	1	1				
β-lactam	3	3	0				
14-macrolide	13	11	3				
15-macrolide	13	10	3				
Quinolones	0	1	0				
Minocycline	2	0	0				
Secondary antibiotics used							
14-macrolide	1	0	0				
15-macrolide	2	7	0				
Quinolones	2	1	0				
Minocycline	26	16	2				
Total duration of fever (days)	7.0 (5.0 – 8.0)	7.0 (5.5 – 8.0)	5.0 (5.0 – 5.5)	p* = 0.04	*P1* = 0.03	*P2* = 0.01	*P3* = ns
Number of patients for whom non-macrolide antibiotics were used as secondary antibiotics	28 (90.3%)	17 (63.0%)	2 (28.6%)	p* = 0.001	*P1* = 0.001	*P2* = ns	*P3* = 0.02
Number of patients requiring hospitalization	1 (3.2%)	1 (3.7%)	0 (0%)	ns			

Data represent medians (1st quantile - 3rd quantile). The Kruskal-Wallis test and Fisher exact test were used to compare the three groups. Wilcoxon test was used to assess pairwise difference.

p*: comparison of A2063G, A2063T and no mutation.

*P1*, *P2*, *P3*: comparison of A2063G vs. no mutation, A2063T vs. no mutation, and A2063G vs. A2063T, respectively.

## Results

### Subject characteristics

During this study period, a total of 105 children were enrolled in this study. The causative pathogens identified were; M.pneumoniae (n = 65, 61.9%), RV (n = 6, 5.7%), HMPV (n = 6, 5.7%), influenza B virus (n = 5, 4.7%), PIV (n = 4, 3.8%), RSV (n = 1, 1%), adeno virus (n = 1, 1%). Mixed viral infections were found in 3 (2.9%) cases. Sixty -five patients (61.9%) with PCR positive for *M. pneumoniae* were analyzed. The patients’ clinical characteristics are shown in Table 1. All patients were outpatients at the time of onset and had no severe underlying illnesses, nor a history of receiving systemic corticosteroids prior to enrollment. Macrolide-resistant mutations in domain V of the 23S rRNA gene were detected in 89.2% (58/65) of the children with *M. pneumoniae* infection, while the remaining 7 (10.8%) were found to harbor no mutation known to be associated with resistance in that region. Thirty-one of the 58 macrolide-resistance mutations (53.5%) showed the A2063G transition; the remaining 27 (46.5%) showed the A2063T transversion in domain V of the 23S rRNA gene. The three groups (A2063G, A2063T, no mutation) did not differ significantly in age, gender distribution or number of days between the onset of initial symptoms and the examination performed (Table 1).

### Patient diagnosis and medication

Detailed information concerning the antimicrobial agents prescribed at the clinic is shown in Table 1. Briefly, fifty-three of 65 (81.5%) patients were initially treated with the 14-membered-ring macrolide CLR or the 15-membered-ring macrolide AZM. In a total of 47 patients (72.3%) these antibiotics were eventually changed to MIN or TFX because of persistent fever, cough, and worsening of chest X-ray findings. A significant difference was observed in the number of patients needing to change to another antimicrobial agent among the three groups (p = 0.001). Specifically, patients with the A2063G transition (90.3%) were more likely to have had the initially prescribed macrolide changed to another antimicrobial agent other than a macrolide in comparison not only to macrolide-sensitive patients (28.6%, p = 0.001) but also to those with A2063T transversion (63.0%, p = 0.02). Fever disappeared dramatically within 48 hr after changing to other drugs such as MIN or TFX in all patients whose clinical findings had failed to improve or whose chest radiographic findings had worsened during the initial treatment. In two patients, symptoms resolved spontaneously without any antibiotics (one with no mutation and the other with A2063T). With regard to the timing of sample collection, 44 of the 65 patients with *M. pneumoniae* had already been treated with antimicrobial agents before nasopharyngeal specimens were collected. There were no statistically significant differences in the incidence of macrolide resistance as to whether sample was collected before or after antimicrobial agents administration (in 17 of 21 (81.0%) collected before administration, and in 41 of 44 (93.2%) collected after administration, p = 0.25).

### Comparison of the clinical courses

The total number of febrile days was significantly greater in patients infected with either of the macrolide-resistance genotypes than in those infected with the no-mutation strains (A2063G: 7.0 days, A2063T: 7.0 days, no mutation: 5.0 days; comparison of A2063G vs no mutation: p = 0.03, A2063T vs no mutation: p = 0.01, and A2063G vs A2063T: not significant). Two patients (one with A2063G and the other with A2063T) required hospitalization because of the progression to dyspnea and the development of a secondary bacterial infection (Table 1).

### Measurement of biologic indicators of inflammation

No significant differences were observed among each genotype group with regard to the results of any acute phase general infection markers (WBC, CRP, ESR). Whereas serum IL-8 and IL-18 levels were not different among the three groups, significant differences were observed in the levels of some serum inflammatory cytokines among the three groups. Serum IFN- gamma, IL-6 and IP-10 levels were significantly higher in patients infected by the macrolide-resistance genotypes than in those infected by the no-mutation strains (comparison of A2063G vs. no mutation: p < 0.001, A2063T vs no mutation: p < 0.001 and A2063G vs A2063T: not significant, Table 1).

### Antimicrobial susceptibility

MICs could be measured exclusively for 15 clinical strains obtained between November and December 2011 (A2063G; 11 strains, A2063T; 4 strains, Table 2) for the reason of performed facilities’ difficulties. Both the A2063G mutation and the A2063T mutation yielded a high degree of resistance to ERY and CLR (MIC range, 64 to >256 μg/mL), though both mutated genotypes exhibited no elevation of MICs for MIN and TFX. Although the 11 strains with the A2063G mutation exhibited variable resistance (MICs 4–64 μg/mL) to the 15-membered macrolide AZM, all 4 strains with the A2063T mutation were susceptible to AZM (MIC 0.25 μg/mL). In addition, we performed the multiple-locus variable-number tandem-repeat analysis (MLVA) for these 15 strains according to the previously described method (18) and found 6 different MLVA types (4 types for the 11 strains of A2063G and other 2 types for the 4 strains of A2063T, data not shown). There was no obvious link between the MLVA type and the patient’s age and residential area, or resistance to macrolides.

**Table 2:** **MICs of selected antimicrobial agents for 15 strains of *M. pneumoniae *** **isolated from clinical samples**

**Point mutation**	**No of strains**	**ERY**	**CLR**	**AZM**	**JOS**	**RKI**	**CLI**	**TET**	**MIN**	**LVX**	**CPX**	**TFX**	**SPX**	**GFX**
A2063G	3	256	> 256	16	16	1	256	1	1	1	1	0.25	0.0625	0.0625
A2063G	2	256	256	16	16	1	256	1	1	1	1	0.25	0.0625	0.0625
A2063G	1	> 256	256	64	16	1	256	1	1	1	1	0.25	0.0625	0.0625
A2063G	1	256	256	16	16	1	128	1	1	1	1	0.25	0.0625	0.0625
A2063G	1	256	256	16	4	1	256	1	1	1	1	0.25	0.0625	0.0625
A2063G	1	256	> 256	8	8	1	256	1	0.25	1	1	0.25	0.0625	0.0625
A2063G	1	256	256	4	4	1	16	1	1	1	1	0.25	0.0625	0.0625
A2063G	1	64	64	4	16	0.5	16	0.5	0.5	1	1	0.25	0.0625	0.0625
A2063T	4	64	64	0.25	16	4	256	1	1	1	1	0.25	0.0625	0.0625

## Discussion

In the present study, we have clarified 89.2% of pediatric *M. pneumoniae* infections were caused by either of two macrolide-resistant genotypes of *M. pneumoniae*. The incidence of MR-*M. pneumoniae* in this general pediatric outpatient setting was nearly as high as those described in continuous surveillance in Japan [4]. Thirty-one of the 58 MR-*M. pneumoniae* strains (53.5%) harbored an A-to-G transition mutation at position 2063 in the 23S rRNA genes, which was consistent with previously reported findings [18] that the A2063G transition was the most frequent mutation. A striking finding in our present work is that the remaining 27 samples (46.5%) showed an A-to-T transversion at position 2063 in the 23S rRNA genes. The A2063T mutation was first reported in China during 2008–2009 [3,19] but has been rarely reported thereafter. However, recent reports from Japan have revealed that the A2063T transversion have been increasing [4,13]. A community outbreak of A2063T transversion was reported recently in Yamagata, Japan, in 2009 [13]. In this point, the resistant strains in our study were polyclonal in origin, which is consistent with the previous report [20]. Although the precise mechanisms of outbreak of the A2063T transversion mutation are not yet known, in general, transversion mutations that change the chemical structure dramatically are thought to affect bacterial growth more severely and in consequence to be less common than transition mutations. For example, an *in*-*vitro* study revealed that the mutation rate of A-to-T transversion is less than one-tenth that of A-to-G transition in *E. coli* cells, coinciding with the spontaneous misinsertion rate of its DNA polymerase III [21].

Another important finding in our study is the profile of serum cytokines and chemokines. It is true that gene amplification methods such as real-time PCR [7] and pyrosequencing [22] must be a best way to detect resistance, however, these methods are confined to very few specialized laboratories in Japan. For reason of that, we expect some additional information on pathogenesis of mycoplasmal infection through measurement of cytokines and chemokines. Higher levels in serum were found for some inflammatory cytokines such as IFN- gamma, IP-10 and IL-6 in patients infected by the macrolide-resistant strains than in those infected by the strains with no-mutation, while no difference was observed in the levels of IL-8 and IL-18 between the two groups. It has been thought IL18 and IL-8 play a pivotal role in the pathogenesis of *M. pneumonia* infection according to the disease severity [23,24]. There are several explanations for this discrepancy. First, disease severity was not so high in our patients infected with macrolide-resistant strains. Indeed we did not experience apparent treatment failure or serious illness. These findings were consistent with previous reports which showed that drug resistance in *M. pneumoniae* does not always lead to severe clinical manifestations [25,26]. Second, the timing of blood sampling might alter the levels of these cytokines as reported by Narita et al. [27], since we only performed blood sampling at the time of enrollment. On the other hand, the higher levels of IFN- gamma, IP-10 and IL-6 in the patients with MR-*M. pneumoniae* infection might reflect, as a non-specific inflammatory marker, the prolonged low degree of inflammation which is represented by slightly longer duration of fever due to the drug resistance. Our results imply the possibility that in addition to the lower clinical efficacy of macrolide treatment, measurement of levels of certain inflammatory cytokines may be a strong candidate that allows the clinicians to suspect MR-*M. pneumoniae* infection.

Strains with the A2063G transition were highly resistant to 14- and 15-membered-ring macrolides as previously described [11,25] and the patients infected by the strains with this mutation were more likely to have had the initially prescribed macrolide changed to a non-macrolide antimicrobial agent when compared with the patients infected not only by macrolide-sensitive strains (p = 0.001) but also by A2063T transversion strains (p = 0.02). As shown by the susceptibility testing, all four strains with the A2063T mutation showed lower MICs for AZM (0.25 μg/mL), and actually, the eight patients with A2063T mutation who were treated with AZM showed complete resolution of clinical symptoms. Thus our data showed that the in-vitro susceptibility directly predicts the clinical efficacy as previously reported [25,26,28]. The reason why AZM is effective in patients with an A2063T transversion is unclear. One plausible explanation is a difference in chemical structures. That is, while the binding of 14-membered-ring macrolides to A2063 is affected both by the G transition and by the T transversion, the binding of 15-membered-ring macrolides to A2063 is not affected by T transversion. Nevertheless, since the A2063T transversion mutation must be related to more profound damage to growth efficiency, the reason remains unknown why so many strains with the rarely reported A2063T mutation, a total of 27, were isolated in this study (in the Fukuoka area). Interestingly, the A2063T mutation slightly but significantly raises the MICs for RKI (4 μg/mL), in contrast to the fact that the strains with the A2063G mutation can be considered susceptible to this macrolide (1 μg/mL). This suggests that A2063G transposition does not affect the binding of RKI to the domain V while A2063T transversion affects the binding to some degree.

The limitations of the present study is that the study was a single-center study conducted over one year; accordingly, the sample number was limited, and the statistical power may therefore have been insufficient to elucidate the linkage between drug susceptibility and genotype of *M. pneumonia*. Further studies including larger sample sizes to investigate the predictive factors of macrolide-resistance are necessary.

## Conclusions

Practical implications of our present study are that the prevalence of MR-*M. pneumoniae* is increasing more rapidly than we thought, even in general outpatient settings and drug susceptibility may be different for each genotype. Moreover, our results reveal the possibility that levels of certain inflammatory cytokines such as IL-6, IP-10 and IFN- gamma may be a candidate to predict MR-*M. pneumoniae* infection at the early stage of infection. In view of these findings, treatment should be tailored to each patient or at least taking into account which patient is infected by the particular genotype, and the treatment strategies must be optimized for each genotype.

## Competing interests

The authors declare that they have no competing interests.

## Authors’ contributions

K Matsuda conceived of the study, participated in its design and coordination, including interpretation of data, and drafted the manuscript. EM, HY and NS participated in the design of the experiments and performed multiplex PCR and analysis of the experiment data. K Matsumoto participated in the design and coordination of the study and performed ELISA immunoassays. HO carried out drug susceptibility test. YA, TK and AF participated in the statistical analysis of clinical data. MN participated in the design and coordination of the study and revision of the manuscript. All authors read and approved the final version of the manuscript.

## Pre-publication history

The pre-publication history for this paper can be accessed here:

http://www.biomedcentral.com/1471-2334/13/591/prepub
